# Case Report: A case of myocardial calcification combined with abnormal Q waves on electrocardiogram

**DOI:** 10.3389/fcvm.2025.1745357

**Published:** 2026-01-26

**Authors:** Dan Li, Qixiang Huang, Xiaoxian Situ, Weidong Gao, Dong Li

**Affiliations:** 1 Department of Electrocardiogram, Jiangmen Central Hospital, Jiangmen, China; 2 Department of Cardiology, Jiangmen Central Hospital, Jiangmen, China; 3 Department of Intensive Care Unit, Jiangmen Central Hospital, Jiangmen, China

**Keywords:** case report, chronic renal failure, dialysis, myocardial calcification, pathological Q wave

## Abstract

**Background:**

Myocardial infarction is the most common cause of pathological Q waves on electrocardiogram (ECG), but other conditions that cause myocardial injury or abnormal conduction (such as cardiomyopathy, myocarditis, cardiac tumors or amyloidosis, WPW, etc.) can also produce pathological Q waves. Whether myocardial calcification can lead to pathological Q waves remains uncertain.

**Case summary:**

A 24-year-old man with a solitary kidney and multiple childhood abdominal surgeries had been on maintenance hemodialysis since 2017 (three times weekly), with a dialysis vintage of approximately 8 years. He was admitted for left upper-limb swelling of 2 months’ duration. On admission his temperature was 36.6°C, blood pressure 93/75 mmHg, and heart rate 95 bpm. ECG showed complete left bundle branch block (cLBBB) and pathological Q waves in leads I, aVL, V5 and V6, without significant ST-T elevation. Transthoracic echocardiography(TTE) revealed global cardiac enlargement with marked systolic and diastolic dysfunction (LVEF ≈ 22%, LVEDD 80 mm), and an irregular hyperechoic mass at the cardiac apex measuring approximately 3.0 × 4.0 cm. Contrast chest CT confirmed focal calcifications in the high lateral wall and a hemispherical calcified lesion at the apex. Laboratory tests showed severe renal impairment (serum creatinine 827 µmol/L), markedly elevated NT-proBNP (32,961 pg/ml), mildly elevated troponin I (0.216 ng/ml) and myoglobin >1,000 ng/ml. Prior records documented severe disturbances of mineral metabolism: serum calcium 1.88 mmol/L, serum phosphorus 3.27 mmol/L, 25-OH vitamin D 13.05 ng/ml, and iPTH up to 2,000 pg/ml.

**Management and outcome:**

The patient underwent diagnostic evaluation, vascular access revision, and continued dialysis; his symptoms improved and he was discharged. He continues regular outpatient hemodialysis and is under follow-up.

**Conclusion:**

We report a rare case of focal myocardial calcification with pathological Q waves in a maintenance dialysis patient. Chronic kidney disease (CKD)-related disturbances of calcium–phosphate metabolism can cause metastatic myocardial calcification. Severe focal calcification may produce mechanical compression and cell necrosis, disrupt electrical coupling, create electrically silent zones, and result in pathological Q waves. In CKD patients with abnormal ECG findings, myocardial calcification should be included in the differential diagnosis and evaluated using imaging and metabolic data.

## Introduction

Myocardial calcifications are uncommon findings, often identified incidentally during imaging or postmortem examinations (1). Myocardial calcification may be categorized as dystrophic calcification following local tissue necrosis or as metastatic calcification secondary to systemic calcium–phosphate metabolic derangement (2, 3). Myocardial infarction (MI) is the most common cause of dystrophic calcification, while chronic kidney disease–mineral and bone disorder (CKD-MBD) is an important cause of metastatic myocardial calcification (2, 3). Myocardial calcification typically manifests as conduction delay or arrhythmia; whether it can directly produce pathological Q waves remains unresolved. We report a maintenance hemodialysis patient with focal apical myocardial calcification accompanied by pathological Q waves, and discuss diagnostic considerations and possible mechanisms.

## Case presentation

### Presenting complaint and history of present illness

A 24-year-old man presented to the interventional department for left upper-limb swelling for 2 months. He denied abdominal pain, nausea, vomiting, chest pain, or dyspnea. On admission his temperature was 36.6°C, blood pressure 93/75 mmHg, heart rate 95 bpm, respiratory rate 20 breaths/min. Physical examination was unremarkable apart from findings related to the dialysis access and cardiac assessment.

### Past medical history

The patient had a solitary kidney, persistent urinary incontinence, and congenital genital hypoplasia. He underwent right nephrectomy at age 3 months, enterostomy at age 8 months, and intestinal surgery with stoma at age 1 year. He began maintenance hemodialysis in 2017 (three times a week) and had a functioning left forearm arteriovenous fistula reconstructed in March 2020.

### Diagnostic assessment (imaging and laboratory tests)

**Figure 1:**
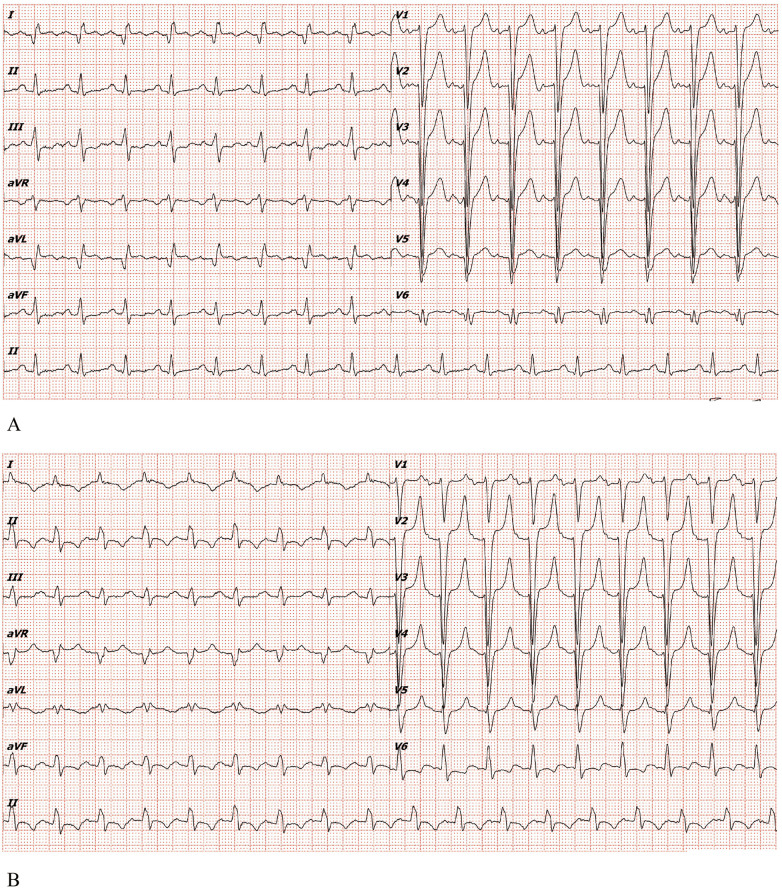
**(A)** Electrocardiogram of the patient in the emergency department of our hospital in 2025. **(B)** Electrocardiogram of the patient in 2022.

Electrocardiogram (ECG) showed complete left bundle branch block (cLBBB) with pathological Q waves in leads I, aVL, V5 and V6, without clear ST-T elevation (Figure 1A). Prior ECGs (Figure 1B) confirmed chronic cLBBB with an rS pattern in V5 and an Rs pattern in V6.

**Figure 2:**
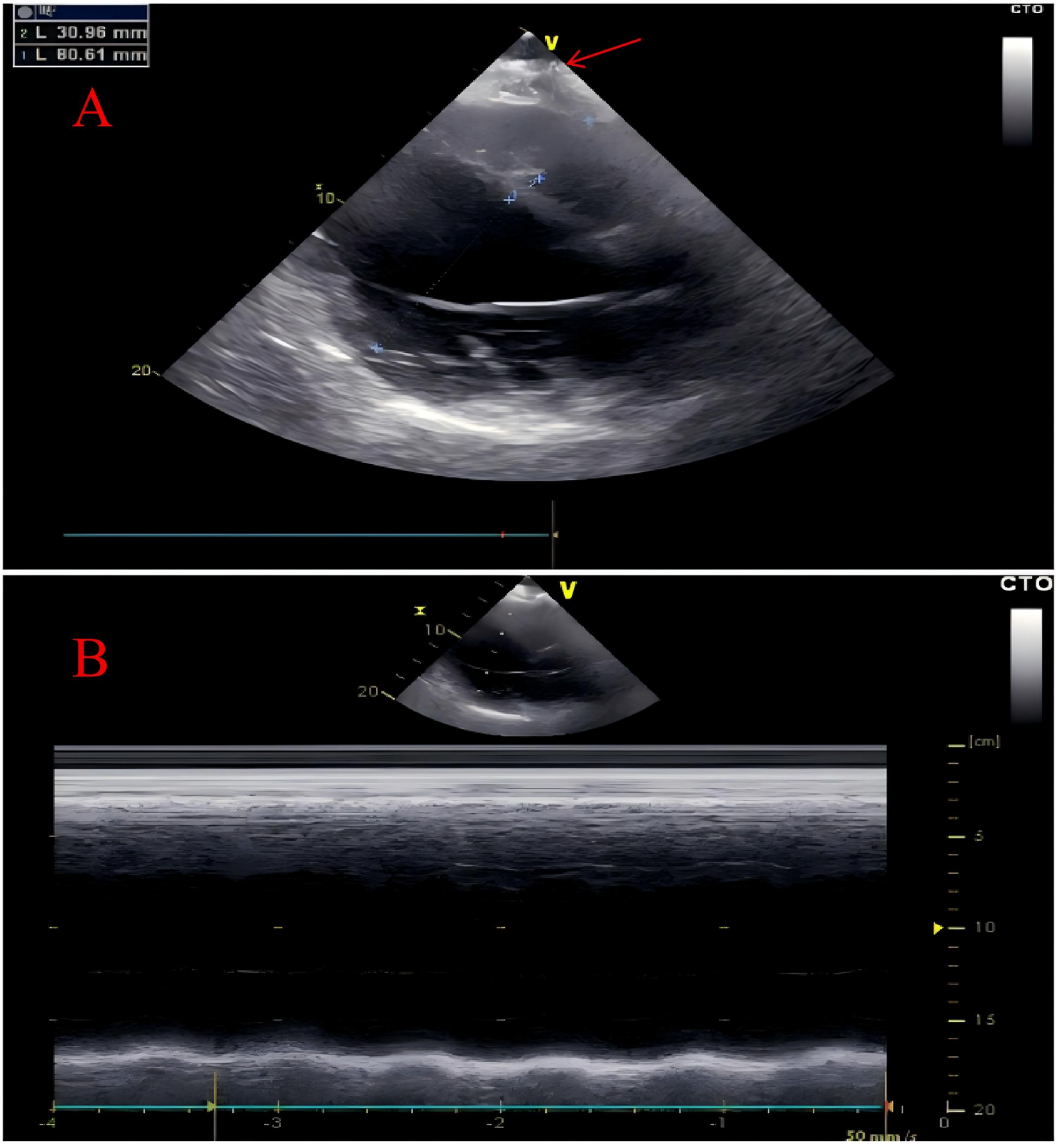
Transthoracic echocardiography. The transthoracic echocardiography revealed an overall enlargement of the heart with calcification at the cardiac apex (marked by red arrow) **(A)** Additionally, the left ventricle demonstrated impaired systolic function **(B)**.

Transthoracic echocardiography (TTE) demonstrated global cardiac enlargement, markedly reduced left ventricular systolic and diastolic function (LVEF ≈ 22%; LVEDD ≈ 80 mm). A linear hyperechoic band was seen in the high lateral wall, and an irregular hyperechoic mass at the apex measuring approximately 3.0 × 4.0 cm with posterior acoustic shadowing suggested myocardial calcification (Figure 2, red arrows).

**Figure 3:**
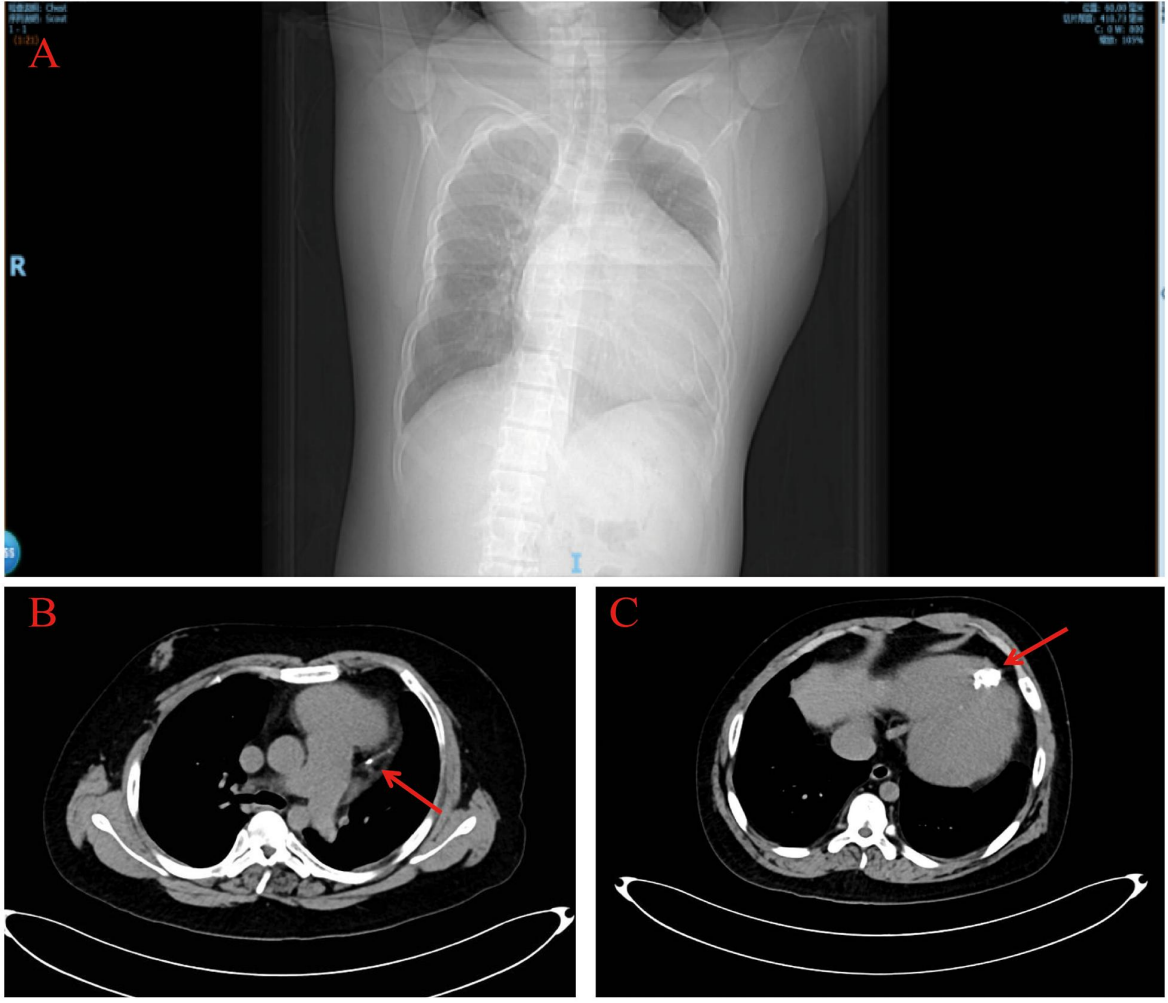
Chest computed tomography. The chest computed tomography revealed a significantly enlarged, spherical heart **(A)** and calcifications at the cardiac apex (red arrows) **(B,C)**.

Chest contrast CT showed a markedly enlarged, globular heart (Figure 3A), linear calcification in the high lateral wall (Figure 3B, red arrow), and a hemispherical calcified lesion at the apex measuring about 3.0 × 4.0 cm (Figure 3C, red arrow).

Laboratory tests: serum creatinine 827 µmol/L (reference 57–97 µmol/L), urea 19.58 mmol/L (3.1–8.0 mmol/L). Cardiac biomarkers: troponin I 0.216 ng/ml (0–0.045 ng/ml), myoglobin >1,000 ng/ml (0–110 ng/ml), CK-MB 2.35 ng/ml (0–5.0 ng/ml), NT-proBNP 32,961 pg/ml (0–125 pg/ml). Infection markers: WBC 7.30 × 10^9^ /L (3.5–9.5 × 10^9^ /L), neutrophils 71.8% (40%–75%), lymphocytes 13.5% (20%–50%), monocytes 11.3% (3%–10%), CRP 16.51 mg/L; blood cultures negative. Historical data showed prolonged mineral metabolism disturbance: serum calcium 1.88 mmol/L (ref 2.11–2.52), serum phosphorus 3.27 mmol/L (ref 0.85–1.15), 25-OH vitamin D 13.05 ng/ml (ref >30), and iPTH ≈ 2,000 pg/ml (ref 18.5–88.0).

## Differential diagnosis

When pathological Q waves coexist with an apical calcified lesion, differential diagnoses include acute or prior myocardial infarction, myocarditis, infiltrative cardiomyopathy (e.g., amyloidosis), cardiac tumors, and metastatic calcification due to systemic metabolic disease. In this case, the absence of typical acute ischemic symptoms, the atypical dynamic pattern of cardiac biomarkers, prior coronary CTA reported without significant stenosis, and imaging evidence of apical calcification favor metastatic myocardial calcification secondary to long-standing CKD-MBD rather than acute MI-related dystrophic calcification.

## Therapeutic intervention

During hospitalization, the patient underwent the described imaging and laboratory evaluations and had vascular access revision. There was no clinical or imaging evidence warranting urgent coronary intervention; prior external coronary CTA showed no obstructive coronary disease, so no acute coronary revascularization or cardiac surgery was performed. After discharge the patient continued regular outpatient hemodialysis and received multidisciplinary management for mineral metabolism (renal and endocrine teams). A timeline of interventions is provided in Table 1.

**Table 1:** Timeline of the case.

Timeline	Events
Date of admission Day 1	A 24-year-old man presented to intervention department of with swelling in his left upper limb for two months, without abdominal pain, nausea, or vomiting, chest pain or shortness of breath. The Electrocardiogram (ECG) revealed complete left bundle branch block (cLBBB) with pathological Q waves in leads I, AVL, V5, and V6, but no arched back elevation with ST-T.
Day 2	Laboratory evaluations revealed abnormal renal function, with creatinine at 827.00 umol/L, Serum troponin I level was 0.216 ng/ml, Myoglobinand level was >1,000 ng/ml, Creatine kinase MBII level was 2.35 ng/ml and NTproBNP level was 32,961 pg/ml. The patient had critical laboratory values, which were considered as metabolic abnormalities caused by renal failure, and the possibility of acute coronary syndrome was excluded.
Day 4	Transthoracic echocardiography (TTE) revealed overall cardiac enlargement with marked systolic and diastolic dysfunction, along with an irregular echogenic mass at the cardiac apex.
Day 5	After regular hemodialysis and related symptomatic treatment, the patient's left upper limb swelling was relieved, so he was discharged and arranged for regular hemodialysis treatment in the outpatient clinic.

## Follow-up and outcomes

Left upper-limb swelling improved. The patient continues routine outpatient hemodialysis and follow-up; during the follow-up period he reported no progressive chest pain or re-admission for acute cardiac decompensation.

## Discussion

This case describes focal apical myocardial calcification with pathological Q waves in a long-term maintenance hemodialysis patient. Key discussion points include: Myocardial calcification results from multiple mechanisms and is classically divided into dystrophic (following local tissue necrosis) and metastatic (from systemic calcium–phosphate imbalance) forms (1). Although dystrophic calcification commonly follows MI, this patient lacked acute ischemic symptoms and biomarker dynamics typical of acute MI. The clinical context—longstanding dialysis, severe hyperphosphatemia, markedly elevated iPTH and low 25-OH-D—supports metastatic calcification due to CKD-MBD as the likely cause.

## Pathophysiological stages toward myocardial calcification in renal failure

Stage 1 (initiation): calcium–phosphate metabolic disorder—impaired renal phosphate excretion leads to hyperphosphatemia; sustained elevation of the calcium–phosphate product provides the biochemical substrate for soft tissue calcification.

Stage 2 (promotion): secondary hyperparathyroidism and altered bone response—uremic bone disease may release large amounts of calcium and phosphorus into the circulation, increasing systemic calcific burden.

Stage 3 (deposition and amplification): loss of calcification inhibitors and local promoting factors—reduced circulating inhibitors and inflammation drive vascular smooth muscle cell osteogenic transformation and active “pathological bone formation,” with myocardial tissue being vulnerable to deposition.

### ECG mechanism of pathological Q waves

Pathological Q waves conventionally indicate transmural myocardial necrosis. We hypothesize that severe focal calcification may produce “pseudo-Q waves” by (1) mechanical compression, fibrosis and cell necrosis creating electrically silent zones, and (2) microvascular/ischaemic microenvironment changes and structural remodeling that reduce depolarization contribution from the affected region, producing prominent Q waves in corresponding leads. Direct histopathological confirmation is lacking, so this mechanism remains speculative.

### Cardiac biomarkers in CKD

Troponins and CK-MB can be elevated in CKD patients in the absence of acute ischemia, partly due to chronic myocardial injury or systemic inflammation, and their elevation correlates with worse prognosis. In this case the biomarker pattern (mild troponin elevation, marked myoglobin rise, non-concordant CK-MB) is more compatible with chronic/metabolic myocardial injury or stress rather than acute extensive myocardial necrosis (4).

### Clinical implications and management

Early recognition and aggressive control of CKD-MBD (phosphate binders, vitamin D analogues, phosphate-lowering strategies, and consideration of parathyroidectomy when indicated) are essential to reduce soft tissue and myocardial calcification risk. Imaging (cardiac CT and echocardiography) is valuable to define extent and morphology of calcification. Patients with severe myocardial calcification or related cardiac dysfunction require multidisciplinary evaluation (cardiology, cardiac surgery, nephrology, endocrinology) to individualize management.

### Limitations

This report lacks myocardial histopathology (no myocardial biopsy) to directly characterize the calcified tissue and cell changes. Systematic long-term imaging follow-up and invasive coronary evaluation were not performed to fully exclude chronic coronary artery disease. Future reports should, when feasible, include tissue diagnosis or more detailed serial imaging to corroborate pathogenesis.

## Conclusion

In maintenance dialysis patients presenting with abnormal ECGs including pathological Q waves, myocardial calcification should be considered in the differential diagnosis. Severe focal myocardial calcification can, under certain conditions, produce localized electrical silence through mechanical and electrophysiological mechanisms, mimicking the ECG appearance of myocardial infarction. Early detection and correction of CKD-MBD are important to prevent or slow metastatic myocardial calcification.

## Data Availability

The original contributions presented in the study are included in the article/Supplementary Material, further inquiries can be directed to the corresponding authors.
